# BAP1 as a guardian of genome stability: implications in human cancer

**DOI:** 10.1038/s12276-023-00979-1

**Published:** 2023-04-03

**Authors:** Jongbum Kwon, Daye Lee, Shin-Ai Lee

**Affiliations:** 1 Department of Life Science, Ewha Womans University, 52 Ewhayeodae-gil, Seodaemun-gu, Seoul, 03760 Korea; 2 Present Address: Laboratory of Genitourinary Cancer Pathogenesis, Center for Cancer Research, National Cancer Institute, Building 37, Room 1068, Bethesda, MD 20892-4263 USA

**Keywords:** Mesothelioma, Double-strand DNA breaks, Nucleotide excision repair, Stalled forks, Mechanisms of disease

## Abstract

BAP1 is a ubiquitin C-terminal hydrolase domain-containing deubiquitinase with a wide array of biological activities. Studies in which advanced sequencing technologies were used have uncovered a link between BAP1 and human cancer. Somatic and germline mutations of the *BAP1* gene have been identified in multiple human cancers, with a particularly high frequency in mesothelioma, uveal melanoma and clear cell renal cell carcinoma. BAP1 cancer syndrome highlights that all carriers of inherited *BAP1*-inactivating mutations develop at least one and often multiple cancers with high penetrance during their lifetime. These findings, together with substantial evidence indicating the involvement of BAP1 in many cancer-related biological activities, strongly suggest that BAP1 functions as a tumor suppressor. Nonetheless, the mechanisms that account for the tumor suppressor function of BAP1 have only begun to be elucidated. Recently, the roles of BAP1 in genome stability and apoptosis have drawn considerable attention, and they are compelling candidates for key mechanistic factors. In this review, we focus on genome stability and summarize the details of the cellular and molecular functions of BAP1 in DNA repair and replication, which are crucial for genome integrity, and discuss the implications for BAP1-associated cancer and relevant therapeutic strategies. We also highlight some unresolved issues and potential future research directions.

## Cancer: Investigating a genome-guarding protein

A protein called BAP1, which removes tags marking proteins for degradation, is an important guardian of genome stability, and understanding its function may help in developing cancer therapies. People with mutations that inactivate BAP1 function always develop at least one and often multiple cancers, but specifically how BAP1 mutations cause cancer is not well understood. Jongbum Kwon and co-workers at Ewha Womans University in Seoul, South Korea, have reviewed how BAP1 functions, focusing on DNA repair. The authors report that BAP1 is crucial for repair of large DNA lesions, such as those caused by UV damage, and plays key roles in DNA replication, chromosome segregation, and disposal of cells with damaged DNA. These activities make BAP1 a powerful tumor suppressor, and improving our understanding of its mechanisms could point the way to new cancer treatments.

## Introduction

BAP1 is a member of the deubiquitinase (DUB) family of proteins and contains a ubiquitin C-terminal hydrolase (UCH) domain^1^. The BAP1 gene, on chromosome 3p21.3, is deleted or mutated in various human cancer cell lines, and re-expression of BAP1 in H226 mesothelioma cells that initially lacked BAP1 expression reversed their tumorigenicity, suggesting that BAP1 might function as a tumor suppressor^1,2^. BAP1 carries a nuclear localization signal (NLS) in the C-terminus and functions in both the nucleus and cytoplasm. The ubiquitin-conjugating enzyme UBE2O monoubiquitinates BAP1 at multiple sites in the NLS, thereby inactivating the NLS to induce cytoplasmic sequestration. The DUB activity of BAP1 counteracts NLS ubiquitination, and this auto-deubiquitination enables BAP1 to be transported to the nucleus, where it executes many biological activities associated with cancer^2–4^. Although BAP1 was originally identified as a protein associated with the BRCA1 tumor suppressor, the significance of the BAP1 and BRCA1 interaction has thus far remained unclear^4–6^.

Several pioneering studies using advanced sequencing technologies have revealed a BAP1 link to human cancer. Inactivating somatic mutations have been identified in *BAP1* in metastasizing uveal melanoma (UM) at a frequency of 84%, with one affected individual carrying a germline mutation^7^. Two human families whose members showed a high incidence of mesothelioma were reported to carry germline *BAP1* mutations and sporadic mesotheliomas in individuals without germline mutations showed somatic truncation mutations of *BAP1* and aberrant BAP1 expression^8^. In addition, 15% of patients with clear cell renal cell carcinomas (ccRCCs) were found to carry inactivating somatic *BAP1* mutations, and some patients carried inactivating germline *BAP1* mutations^9^. Subsequently, numerous studies confirmed a link between *BAP1* germline mutations and a predisposition to mesothelioma^10–14^, UM^15,16^ and ccRCC^17,18^ as well as to other cancer types, such as cutaneous melanoma^19^ and basal cell carcinoma^20,21^. These findings have led to the proposal of BAP1 cancer syndrome, which describes the case in which carriers of inherited *BAP1*-inactivating mutations develop at least one and often multiple cancers during their lifetime, with the overall penetrance approaching 100%^22^. In the case of mesothelioma, although *BAP1* germline mutations induce spontaneous cancer development, cancer incidence increases upon exposure to asbestos, a carcinogenic fiber that is closely associated with mesothelioma, providing an excellent example to study how gene-environment interactions influence cancer risk^23–27^.

In human cells, BAP1 is expressed in a multiprotein complex comprising as many as ten different subunits, including Additional sex combs-like 1 (ASXL1), 2 and 3, human homologs of the *Drosophila* Polycomb group protein ASX, which associates with different assemblages of the BAP1 complex in a mutually exclusive manner^28–32^. *Drosophila* cells contain a similar complex named Polycomb repressive deubiquitinase (PR-DUB), consisting of Calypso, which is closely related to the human BAP1 homolog, and ASX^33^. BAP1 and PR-DUB remove ubiquitin from H2A-K119-ub and H2A-K118-ub (both denoted H2Aub hereafter), respectively, which are the transcriptional histone markers catalyzed by the Polycomb (PcG) group complex PRC1^32,33^. BAP1 participates in a wide range of biological processes by directly targeting proteins as substrates or indirectly via transcription, and these BAP1-regulated processes include cell cycle control^28–30,34,35^, cell survival and proliferation^32,36,37^, cell death^38–43^, the DNA damage response (DDR) and repair^44–47^, DNA replication^48–51^, metabolism^39,52–54^, and cell differentiation and development^36,55–57^. For example, BAP1 promotes apoptosis by deubiquitinating and stabilizing IP3R3 at the endoplasmic reticulum (ER), which stimulates Ca^2+^ release from the ER into the cytosol and thereby increases the mitochondrial Ca^2+^ concentration and cytochrome C release^41^. BAP1 also indirectly regulates apoptosis and ferroptosis, a recently identified nonapoptotic form of cell death, by regulating the transcription of the genes critical for these processes^39^.

Gene-targeting studies have documented that *BAP1* is essential for embryogenesis, and conditional disruption of *BAP1* in the hematopoietic lineage and kidney of adult mice led to the development of myeloid neoplasia and ccRCC, respectively^55,58,59^. In addition, mice carrying heterozygous germline *BAP1* mutations developed various spontaneous tumors and were predisposed to the development of malignant mesothelioma after exposure to asbestos carcinogenic fibers^25–27^. These studies, together with those on *BAP1* mutations in human cancers and the roles of BAP1 in many cancer-related biological activities, strongly suggest that BAP1 functions as a tumor suppressor. Nonetheless, the mechanisms underlying the tumor suppressor function of BAP1 have only begun to be uncovered. While many BAP1 activities are likely involved in tumor suppression, two important mechanisms have recently drawn considerable attention: genome stability and apoptosis. In this review, we summarize the details of the currently known cellular and molecular functions of BAP1 in DNA repair and replication, focusing on genome stability, and we discuss the implications of these functions for BAP1-associated cancer and potential therapeutic strategies. We also highlight some unsolved issues and provide perspectives for future research directions. We refer to excellent recent reviews on the roles of BAP1 in apoptosis and in other biological processes^4,22,24,31^.

## The role of BAP1 in double-strand break (DSB) repair

### The DSB repair pathway

Double-strand breaks (DSBs) can be generated by exogenous agents, such as ionizing radiation (IR), and can be generated endogenously by the collapse of replication forks that encounter various obstacles, such as ultraviolet (UV)-induced DNA lesions. The two major pathways for DSB repair in mammalian cells are homologous recombination (HR) and nonhomologous end joining (NHEJ)^60^. During HR, the MRN (Mre11/Rad50/Nbs1) complex recognizes and binds the ends of DNA breaks and generates a 3’ single-stranded DNA tail via 5′-to-3′ strand resection^61^. After rapid coating with replication protein A (RPA), the emergent single-stranded DNA (ssDNA) is bound by RAD51, resulting in the formation of a nucleoprotein filament. The RAD51-ssDNA nucleoprotein filament then searches nearby homologous sequences facilitated by the BRCA1-BARD heterodimer and BRCA2, leading to the formation of a double Holliday junction. Subsequent migration and resolution of the Holliday junction completes the recombination reaction to repair DSBs in an error-free fashion^62^. NHEJ is initiated with binding of the Ku70-Ku80 heterodimer to a DSB end, which facilitates the recruitment of other factors, including the DNA-dependent protein kinase catalytic subunit (DNA-PKcs), the Artemis nuclease, and DNA ligase IV, which is associated with XRCC4; this recruitment leads to nucleolytic processing and DNA ends being directly joined and is typically accompanied by a few base pair insertions or deletions^63^. A series of control mechanisms determines whether a DSB is repaired via HR or NHEJ, and these mechanisms depend on the cell cycle phase and the local chromatin environment^60^.

**Fig. 1:**
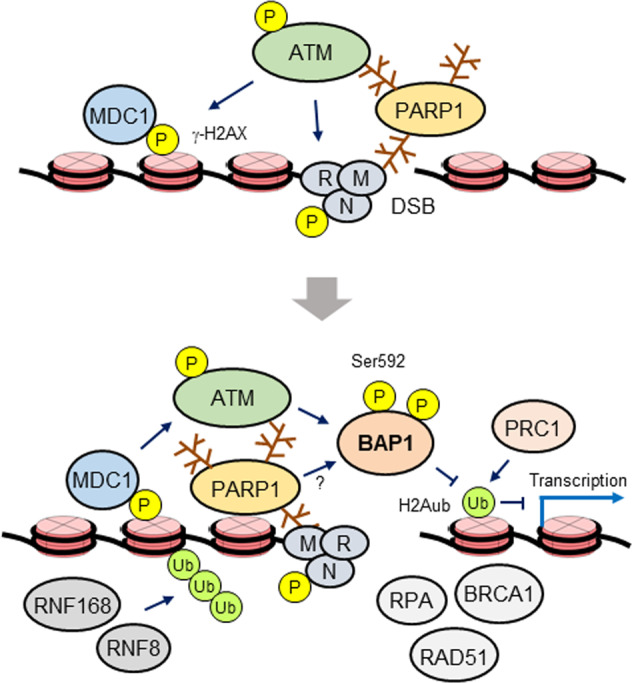
Model showing the role for BAP1 in DSB repair. After binding to DSBs, the MRN damage sensor recruits and activates ATM by triggering autophosphorylation, leading to the phosphorylation of H2AX and MDC1. Then, more ATM-MRN complexes are recruited through a positive feedback loop, which activates RNF6/168-mediated ubiquitin signaling, leading to the recruitment of HR proteins, including BRCA1 and RAD51. ATM phosphorylates BAP1 at multiple sites, including Ser592, which triggers BAP1 recruitment to DSBs via an unknown mechanism. PARP1/2 and RNF6/168 also mediate BAP1 recruitment. The exact role played by BAP1 in DNA repair is unclear. BAP1 might promote DNA repair by recruiting HR proteins, such as BRCA1, RAD51 and RPA, and/or by regulating the levels of H2Aub, which is enriched at DSBs, in cooperation with the RNF2 H2A E3 ligase (in a PRC1 complex). To determine whether PARP1 regulates BAP1 activity during DSB repair, more investigation is needed.

Cells respond to DSB generation by activating the DDR, which involves a complex signaling network that coordinates damage checkpoint activation, cell cycle arrest, DNA repair and, in some cases, apoptosis. The signaling cascades that direct the DDR during HR have been extensively characterized. ATM kinase plays a central role in initiating the DDR. After recruitment to DSBs via its interaction with MRN, ATM phosphorylates and activates multiple DDR proteins, including ATM itself and histone H2A variant X (H2AX). Phosphorylated H2AX (γ-H2AX)^64^ recruits adaptor DNA damage checkpoint 1 (MDC1) to a DSB, leading to the recruitment and binding of more MRN-ATM complexes. PARP1, the predominant and initially discovered member of the PARP family, which cleaves nicotinamide adenine dinucleotide (NAD^+^) to ADP-ribose and nicotinamide and covalently attaches ADP-ribose units to target proteins, including itself, forming a linear or branched poly(ADP-ribose) (PAR) chain, independently binds to DSBs and recruits ATM and MRN via PAR-mediated interactions^65,66^. The newly recruited ATM phosphorylates proximal H2AX, which then serves as a platform to recruit MRN-ATM complexes, forming a specialized chromatin structure extending megabase away from the DSB^67,68^. ATM-phosphorylated MDC1 recruits RNF8, resulting in the formation of ubiquitin chains on the H2A surrounding a DSB. RNF168 binds to these ubiquitinated histones and catalyzes Lys63-linked polyubiquitination of H2A, which recruits key downstream factors, such as BRCA1 and RAD51^69,70^. PRC1 also catalyzes the monoubiquitination of H2A at K119 to facilitate DNA repair by blocking the transcription of genes near the DSB^71^. γ-H2AX additionally recruits chromatin-remodeling complexes, such as INO80 and SWI/SNF, to facilitate DNA repair^67^ (Fig. 1).

### The role of BAP1 in DSB repair

Motivated by the evidence for the potential involvement of DUBs in DSB signaling and repair^72^, the Affar group and colleagues sought to identify DUBs required for the recruitment or dispersion of repair proteins at IR-induced repair foci. Using a functional RNAi screening approach, the authors identified BAP1 as a DUB required for efficient assembly of RAD51 or BRCA1 after IR. BAP1 bound to chromatin in response to IR and was recruited to a DSB that had been induced by the I-SceI endonuclease on reporter DNA. BAP1 depletion reduced the quantity of both RAD51 and BRCA1 foci without affecting the respective protein levels and exerted no effect on the formation of 53BP1 or autophosphorylated DNA-PK foci, suggesting that BAP1 functions in HR but not in NHEJ^44^. The authors confirmed the HR activity of BAP1 by showing that DT40 chicken B-lymphoma cells exhibited increased IR sensitivity and chromosomal aberrations after ablation of both *BAP1* alleles^44^. Notably, BAP1-ablated DT40 cells were highly sensitive to the poly(ADP-ribose) polymerase (PARP) inhibitor olaparib, further corroborating the HR activity of BAP1, as cancer cells with inactivating mutations in BRCA1 or BRCA2, which are thus deficient in HR, were found to be hypersensitive to PARP inhibition^73–75^. These results were consistent with a report indicating that BAP1 loss sensitized ccRCC cells to IR and olaparib^9^ (Fig. 1).

Hendzel and colleagues independently demonstrated the role of BAP1 in HR^46^. The authors showed that BAP1, together with γ-H2AX, accumulates rapidly and transiently at laser microirradiation-induced damage sites and is recruited to DSBs induced by a FokI endonuclease on reporter DNA. However, BAP1 did not form clear foci after IR but showed enhanced resistance to detergent extraction from chromatin. This finding suggested that BAP1 was unlikely to be translocated to damage sites globally; in contrast, the affinity of BAP1 to DNA lesions increased. PARP1/2 and RNF8/168, but not γ-H2AX or ATM, were required for BAP1 recruitment to damage sites, although the mechanisms remained unknown. Then, by using a GFP-based reporter assay, the authors showed that BAP1 promoted HR in a BRCA1-related pathway without exerting a significant impact on NHEJ. Consistently, BAP1 depletion, which increased IR sensitivity, reduced the quantity of foci of HR factors, such as BRCA1, RPA and RAD51 but did not inhibit the formation of foci of NHEJ factors, such as 53BP1 and BMI1^46^. The catalytic activity of BAP1 was shown to be important to damage site recruitment and DSB repair^44,46^ (Fig. 1). A recent study confirmed the activity of BAP1 in HR on the basis of a GFP-based reporter assay; loss of BAP1 caused a substantial reduction in the DNA repair rate in pancreatic cancer cells^76^. Increased IR sensitivity after BAP1 depletion has also been observed in HeLa and ccRCC cells^5,9^.

Evidence suggests that BAP1 phosphorylation is important for DSB repair. A large-scale proteomic analysis identified BAP1 phosphorylation at Ser592, an SQ/TQ motif in ATM/ATR kinase consensus phosphorylation site, after IR^77^. Then, immunoprecipitation of BAP1 combined with a mass spectrometry (MS) analysis revealed IR-induced phosphorylation at several sites, including Ser592. A BAP1 mutant in which all identified phosphosites were replaced with Ala residues did not support DSB repair or cell survival after IR and was not enriched at an I-SceI-induced DSB^44^. The combination of mutations in two identified SQ motifs only partially reduced BAP recruitment to a DSB^44^, and individual mutation of all predicted SQ/TQ motifs, including Ser592, did not interfere with BAP1 recruitment to laser microirradiation-induced damage sites^46^. The BAP1 phosphorylation mutant exhibited no apparent defects in protein complex assembly or nucleosomal H2A deubiquitination in vitro^44^. Therefore, it appeared that multiple phosphorylation events act together to promote BAP1 recruitment and DNA repair independent of its catalytic or complex-assembly activities (Fig. 1).

### Unsolved problems and potential directions for future research

In addition to the mechanisms underlying BAP1 damage-site recruitment, the way in which BAP1 promotes DSB repair remains largely unknown. Several mechanisms might provide some answers. Since BAP1 is required for the accumulation of BRCA1, RAD51 and RPA at damage sites^44,46^, BAP1 may facilitate DSB repair by recruiting these factors directly via protein-protein interactions. This mechanism is plausible, at least for BRCA1, given that BAP1 directly interacts with this repair protein^1,5^. The RING domain of BRCA1, which engages in E3 ubiquitin ligase activity, is important for its HR-promoting activity^78,79^. Thus, BAP1 may promote DSB repair by regulating the deubiquitination of BRCA1 substrates and even BRCA1 itself. H2Aub is important for the RNF8/RNF168-mediated ubiquitin signaling cascade that leads to the recruitment of repair factors, including BRCA1^67^. PRC1-mediated H2A ubiquitination facilitates DSB repair by blocking the transcription of regions adjacent to the damage site^80^. Therefore, BAP1 may contribute to DNA repair by regulating H2Aub levels at damage sites. This scenario is possible because H2Aub levels at a DSB are inversely correlated with BAP1 recruitment^44^, and BAP1 depletion increases H2Aub accumulation at a DSB^46^ (Fig. 1). However, given the general consensus that BAP1-mediated H2A deubiquitination activates transcription^43,71,81^, how BAP1 promotes HR repair via H2Aub regulation remains unclear. BAP1 might regulate H2Aub abundance at chromatin surrounding damage sites to achieve an optimal balance between the activities of DNA repair and transcription. Alternatively, BAP1 might differentially affect DNA repair at transcriptionally active or inactive and intergenic regions. Another plausible explanation suggests that BAP1 might facilitate the whole repair process by promoting transcription recovery after activation and/or completion of DNA repair. In addition, the exact role of BAP1 phosphorylation in DSB repair remains unknown and certainly needs to be clarified. Moreover, given the recent findings that PARP1 recruits BAP1 to damage sites and regulates BAP1 activity during UV-induced DNA damage repair (see below)^47^, determining whether PARP1 shows similar activity in DSB repair is worthy of further investigation (Fig. 1). Finally, since studies have indicated that BAP1 regulates the expression of genes encoding DDR proteins^30,34^, an indirect contribution by BAP1 to DSB repair via regulated gene expression cannot be ruled out. Resolving all these issues, together with efforts to identify potential new BAP1 targets, will provide a better understanding of how BAP1 promotes DSB repair.

## The role of BAP1 in nucleotide excision repair (NER)

### The NER pathway

The NER pathway repairs a wide range of structurally unrelated bulky DNA lesions, including those induced by UV exposure, such as cyclobutane-pyrimidine dimers (CPDs). Two subpathways mediate NER, and the early steps in these pathways involve activation of different mechanisms of damage recognition. The global genome NER (GG-NER), the dominant subpathway of NER, surveys the entire genome for helix distortions via the damage-sensing protein xeroderma pigmentosum C (XPC) as well as the UV-damaged DNA binding (UV-DDB) complex that comprises DDB1 and DDB2. The transcription-coupled NER (TC-NER) subpathway rapidly removes transcription-blocking lesions via the recognition of stalled RNA polymerase II with the Cockayne syndrome A (CSA) and B (CSB) proteins. Following damage recognition, these two subpathways converge, thereafter following the same pathway, which involves removal of damaged DNA via double incision, followed by synthesis of a new stretch of nucleotides and, ultimately, DNA ligation^82,83^.

**Fig. 2:**
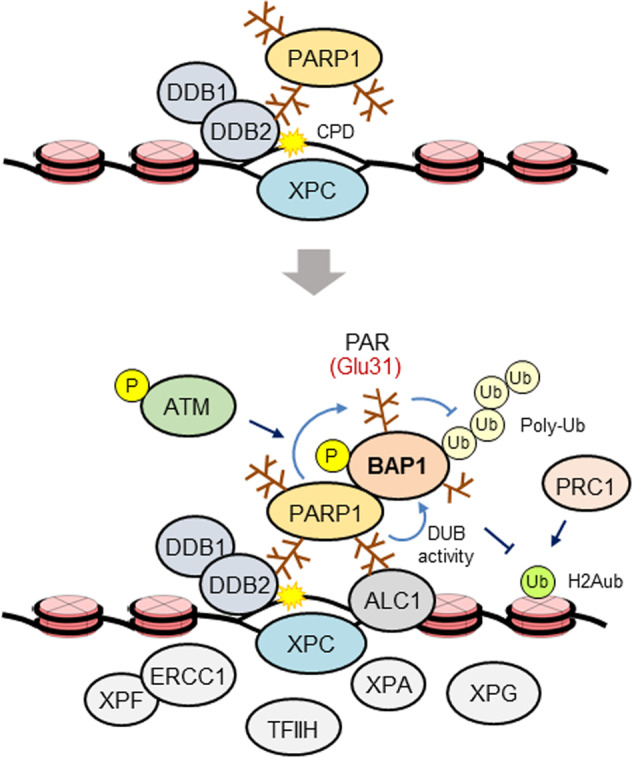
Model showing the role played by BAP1 in NER. DDB1/2 and XPC together recognize CPDs and accumulate rapidly at the site of these lesions. PARP1 is recruited early to lesions independent of damage sensor activity. Both PARP1 and H2Aub, which accumulate at lesions, mediate BAP1 recruitment, with PARP1 directly engaging in protein-protein interactions independent of PARylation. PARP1 also activates BAP1 both intrinsically and via PARylation to stimulate DNA repair. PARP1 PARylates BAP1 at multiple sites, including Glu31, which is mutated with high frequency in ccRCC, and Glu31 stabilizes BAP1 by inhibiting degradative ubiquitination that is mediated via crosstalk between the PARylation and ubiquitination machinery. How BAP1 promotes CPD repair is unknown. As postulated for HR, BAP1 might promote DNA repair by regulating H2Aub levels at lesions given the importance of H2Aub in NER. ATM phosphorylates BAP1 at Ser592 after UV irradiation, but the effect of this event is unknown.

In the GG-NER, XPC constantly surveys DNA for helix-distorting lesions and binds to ssDNA opposite a lesion facilitated by the UV-DDB complex, which stabilizes XPC by directly binding to the lesion^84–86^ (Fig. 2). In the TC-NER subpathway, CSA and CSB indirectly recognize damage by binding to RNA polymerase II when it is stalled at a lesion during transcription elongation^87^. After damage recognition, the TFIIH complex, a transcription initiation and repair factor with DNA helicase activity, is recruited to the lesion in both GG-NER and TC-NER subpathway^88^. The TFIIH complex verifies the lesion by extending the open DNA configuration around the lesion, which is stabilized by the binding of the ssDNA-binding protein XPA^86^. Subsequently, the endonucleases XPF-ERCC1 and XPG are recruited to the lesion and incise the damaged strand at short distances 5′ and 3′ from the lesion, respectively, leading to the removal of a 22–30 nucleotide-long ssDNA sequence that includes the lesion. The DNA replication machinery and DNA ligase execute gap-filling DNA synthesis and final nick sealing to complete NER^82^ (Fig. 2).

PARP1 plays an important role in the initial steps of damage recognition in the GG-NER subpathway^89^. Upon UV damage, PARP1 rapidly localizes to lesions independent of the UV-DDB complex, and this localization stimulates PARP1 catalytic activity, leading to PARylation of itself (auto-PARylation), DDB2 and other target proteins. PARylation stabilizes DDB2, and DDB2 interacts with PARP1 and stimulates PARP1 catalytic activity, which facilitates its recruitment to a damage site and subsequent stabilization of XPC, which binds to PAR through its own PAR-binding site^90^. In addition, DDB2-stimulated PARP1 PARylates histones, leading to recruitment of the ATP-dependent chromatin remodeling protein ALC1 via its own PAR-binding domain, which further stimulates DNA repair through the reconfiguration of nucleosomes surrounding the lesion^91^ (Fig. 2).

### The role of BAP1 in NER

Given the implications of a role played by H2Aub in NER^92–94^, one may postulate that BAP1 might play a role in NER as a regulator of H2Aub. Indeed, after UV irradiation, BAP1 is phosphorylated at multiple sites, including at Ser592^45,95^. Recently, Carbone and colleagues subjected fibroblasts derived from *BAP1*^+/-^ carriers and from their age- and sex-matched wild-type *BAP1* family members, who served as controls, to UV radiation and compared the DNA repair activity level by evaluating the level of γ-H2AX, the cellular indicator of a DSB^64^. They observed that compared to wild-type fibroblasts, *BAP1*^+/-^ fibroblasts exhibited a prolonged γ-H2AX response, suggesting that a reduction in BAP1 levels might have impaired the repair of UV-induced DNA damage, leading to the production of secondary DSBs via replication fork collapse^41^. These studies, however, did not provide direct evidence for the involvement of BAP1 in the repair of UV-induced DNA damage.

Kwon and colleagues directly investigated whether BAP1 functions in NER and found that BAP1 depletion in HEK 293 T cells resulted in defective CPD repair and increased sensitivity after UV irradiation and that BAP1, but not the C91S catalytic mutant, rescued the repair defects in BAP1-depleted cells. BAP1-dependent CPD repair was then observed in various other cell types, including U2OS osteosarcoma cells, KMRC20 ccRCC cells and human primary epithelial melanocytes. BAP1 bound chromatin and formed foci overlapping with CPDs immediately after UV irradiation, with BAP1 chromatin binding and foci formation peaking 30 and 60 min after exposure^47^. These results suggested that BAP1 directly promotes CPD repair via its catalytic activity and that this role of BAP1 is not cell-type specific. BAP1 did not appear to participate in damage recognition since BAP1 recruitment to the lesions peaked after DDB2 and XPC recruitment, exhibiting a very rapid response to UV irradiation^90,96^, and BAP1 depletion did not affect DDB2 or XPC recruitment^47^ (Fig. 2).

The mechanisms underlying BAP1 recruitment to damage sites involve H2Aub and PARP1. H2Aub formed clear foci overlapping with CPDs immediately after UV irradiation, and depletion of Ring1A and Ring1B, the major E3 ligases of H2A, greatly reduced the quantities of both H2Aub and BAP1 foci accompanied by CPD repair defects, indicating that H2Aub is important for BAP1 recruitment^47^. PARP1 interacted with BAP1 after UV irradiation, and depletion of PARP1 or treatment with PARP inhibitors reduced BAP1 chromatin binding and BAP1 colocalization with CPDs. In addition, GFP-PARP1 accumulated at damage sites faster than GFP-BAP1 after laser microirradiation in a live cell analysis. Importantly, a BAP1 mutant lacking UV-induced PARP1-binding activity did not rescue the repair defects in BAP1-depleted cells^47^. These results demonstrated that PARP1 recruits BAP1 to sites of UV-induced DNA damage. However, the role of H2Aub in BAP1 recruitment may be complex and challenging to understand, as BAP1 removes ubiquitin from H2A. One possible scenario suggests that after recruitment to damage sites via H2Aub, BAP1 targets H2Aub and fine-tunes the H2Aub level to regulate its own recruitment and that of other repair proteins (Fig. 2).

The authors further showed that PARP1 regulates BAP1 activity in addition to mediating BAP1 recruitment to damage sites. In vivo PARylation assays showed that PARP1 PARylated BAP1 and that this PARylation was constitutively activated and increased transiently after UV irradiation. PARylation was not required for the interaction between BAP1 and PARP1 since treatment with PARP inhibitors did not interfere with this interaction^47^. This result, which is consistent with the fact that BAP1 does not directly interact with the PAR polymer^46^, indicated that BAP1 recruitment to damage sites was independent of its PARylation. A series of in vitro studies showed that PARP1 stimulated BAP1 activity toward Ub-AMC artificial substrates and that PARylation further enhanced this activity. Interestingly, PARP1 stimulated BAP1 activity toward the physiological substrates of H2Aub that had assembled into a nucleosome, but PARylation completely inhibited this activity and was accompanied by strong BAP1 binding to the nucleosomes. It was proposed that although PARP1 stimulates BAP1 both intrinsically and via PARylation, probably via an allosteric mechanism, PARylated BAP1 formed an unproductive complex with H2A-ub nucleosomes, rendering H2Aub untargetable^47^. Although the mechanisms and functional significance remain unclear, the differential activities of PARP1 toward BAP1 on Ub-AMC and H2Aub nucleosomes may reflect the complexity of their control over CPD repair in the context of chromatin substrates within cells (Fig. 2).

In vitro BAP1 PARylation combined with MS enabled the identification of multiple sites that were PARylated, and many of these sites were mutated in various human cancers. Among these cancer mutations, Glu31, which was particularly frequently mutated in ccRCC, was shown to promote BAP1 stability via crosstalk between PARylation and ubiquitination machinery^47^. The PAR chains on Glu31 may recruit PAR-dependent E3 ligases to BAP1 for proteasomal degradation^97^. This finding led to the addition of BAP1 to the list of proteins with stability that is controlled by crosstalk between PARylation and ubiquitination machinery^66^. Intriguingly, a BAP1 mutant at Ala31 (a non-PARylatable residue) did not rescue CPD repair in BAP1-depleted cells even when expressed at normal levels, suggesting that PARylation at Glu31 plays an additional role in CPD repair in addition to protein stabilization. Glu31 also participates in the reduced viability of ccRCC cells, likely reflecting its tumor suppressor activity, seemingly via mechanisms independent of DNA repair (Fig. 2).

Compared to a *BAP1* wild-type control, fibroblasts carrying a heterozygous *BAP1* mutation accumulated more DNA damage after UV exposure due to their reduced DNA repair ability; however, these mutant cells were resistant to apoptosis owing to a decreased IP3R3 level, resulting in increased cell survival even after DNA was damaged^41^. These results suggest that a decrease in BAP1 levels may contribute to cell transformation by causing increased DNA damage and reduced apoptosis. Therefore, the finding that BAP1 promotes the repair of UV-damaged DNA in primary melanocytes may explain why cutaneous melanomas and skin cancers—often caused by UV radiation—are prevalent in carriers of germline *BAP1* mutations^47^.

### Unsolved problems and potential directions for future research

The study from the Kwon group, demonstrating the role of BAP1 in NER for the first time, raised some important mechanism-specific and many new questions. The first and most important question is how does BAP1 stimulate CPD repair? Notably, factors targeted by BAP1 at DNA lesions, if they exist, remain to be discovered, and how BAP1 targeting affects CPD repair needs to be determined. It is also important to determine the role H2Aub played in CPD repair and how BAP1 regulates H2Aub levels at DNA lesions (Fig. 2). Second, what are the precise mechanisms underlying the recruitment of BAP1 to damage sites? Although both H2Aub and PARP1 are involved in BAP1 recruitment, whether these factors work independently or act in cooperation is unknown. Thus, among questions, it will be interesting to know whether H2Aub promotes PARP1 recruitment and/or whether PARP1 positively regulates H2Aub enrichment at damage sites. In addition, many important questions regarding the cellular and molecular functions of PARP1 activity toward BAP1 remain unanswered. These questions include (1) Does PARP1 stimulate BAP1 within cells, and how is this activity regulated during CPD repair? (2) Whether and to what extent is BAP1 PARylated at each of the identified PARylation sites within cells? (3) What are the functions of BAP1 PARylation sites, in addition to that of Glu31, particularly those that are mutated in human cancers, in CPD repair and possibly other cancer-associated cellular processes, such as cell death and genome stability? (4) Does PARP1-mediated BAP1 PARylation exert an effect on DSB repair in which PARP1 plays an important role^89^. Finally, since BAP1 possesses both deubiquitinating activity and the activity to recruit cellular factors through the formation of multiprotein complexes and/or association with other proteins, which might act either independently or in concert in different cellular processes^6^, it will be of great interest to investigate whether and how BAP1 might differentially use these two activities in HR-mediated DSB repair and NER.

## The role of BAP1 in DNA replication

### DNA replication

**Fig. 3:**
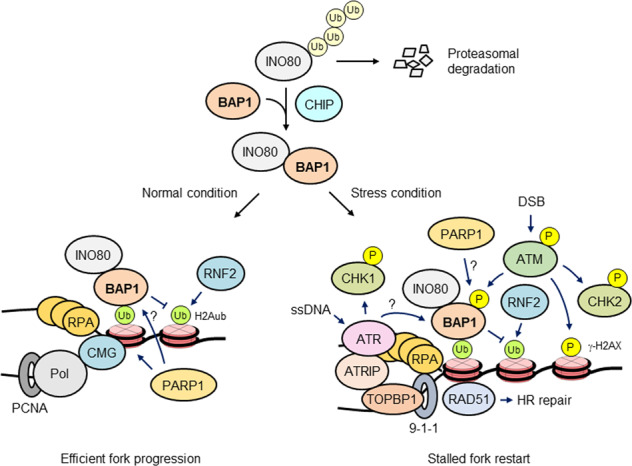
Model showing the role of BAP1 in DNA replication. BAP1 binds, deubiquitinates and stabilizes INO80 by preventing its ubiquitin-mediated proteasomal degradation. The CHIP E3 ubiquitin ligase cooperates with BAP1 to reinforce INO80 stabilization, probably by replacing the degradation-signaling polyubiquitin chain on INO80 with a nondegradation-associated chain. BAP1 recruits INO80 to replication forks, where INO80 promotes fork progression during normal DNA synthesis. BAP1 recruitment to replication forks is mediated by its interaction with H2Aub, which is enriched at forks by the E3 ligase activity of RNF2 (in an RRC1 complex). BAP1/INO80 recruitment to replication forks via H2Aub is increased during replication stress and leads to RAD51 recruitment and stalled forks restart. INO80 presumably increases DNA accessibility by modulating chromatin structure upstream of the replication fork and/or restores prereplication chromatin downstream of the fork as a way to promote fork progression and stalled fork restart. ATM phosphorylates BAP1 at Ser592 after HU treatment, but the role played by this modification is unknown. Whether ATR targets BAP1 during replication stress needs to be investigated. BAP1 might play a direct role in DNA replication, for example, by regulating H2Aub at replication forks. Although PARP1 participates in both normal and stressed DNA replication, whether PARP1 plays a role via BAP1 action remains to be elucidated.

DNA replication is the key process in the cell division cycle and entails making an exact copy of the genome and transmitting only one complete genome set to each daughter cell. A multiprotein molecular machine, known as the replisome, comprising DNA polymerase associated with the PCNA sliding clamp and numerous auxiliary factors, such as the replicative helicase CMG (CDC45-MCM2-7-GINS) and the RPA ssDNA-binding protein, executes this vitally essential nuclear process. Efficient DNA replication requires ATP-dependent chromatin-remodeling enzymes, such as INO80 and the SMARCAD1 SWI/SNF-like remodeling factor, which ensure precise copying of the epigenetic code during replication and restoration of the chromatin configuration after replication. Histone modifications, such as acetylation, methylation, ubiquitination and ADP-ribosylation, also contribute to efficient fork progression and the maintenance of the epigenome landscape after replication by promoting nucleosome assembly, chromatin structure reorganization and replication factor recruitment (Fig. 3)^98,99^.

DNA replication is often challenged by numerous endogenous or exogenous obstacles, such as nucleotide shortages, DNA lesions and secondary DNA structures, and by its inevitable encounters with transcription machinery. These challenges during DNA replication can produce replication stress, a complex phenomenon characterized by slowed DNA synthesis and replication fork stalling and/or collapse accompanied by DNA breaks. Cells respond to replication stress by activating S-phase checkpoints and delaying cell cycle progression, thereby providing time for stabilization, stalled fork restart and DNA repair in cases of fork collapse^100,101^. Stalled replication forks lead to the accumulation of extended ssDNA sequences, to which RPA binds and recruits ATM and Rad3-Related (ATR), a master kinase, together with ATM, which is central to the HR-mediated DSB response, through its partner ATR-interacting protein (ATRIP). ATR is activated by DNA topoisomerase 2-binding protein 1 (TOPBP1), which is recruited by the 9-1-1 complex, a PCNA-like clamp consisting of RAD9, RAD1 and HUS1. ATR then phosphorylates and activates checkpoint kinase 1 (CHK1), which triggers the ATR-CHK1 pathway, leading to cell cycle arrest, replication fork stabilization and the activation of DSB repair through the recruitment of HR factors, such as RAD51. Collapsed replication forks often generate fork reversal and thus DSBs, which recruit and activate ATM, leading to the activation of several targets through phosphorylation, such as H2AX and CHK2. ATM, in cooperation with ATR, orchestrates signaling events that promote cell cycle checkpoints and HR-mediated DSB repair, ensuring replication fork stabilization and restart^98,102,103^. PARP1 not only participates in unperturbed DNA replication but also plays a crucial role in fork stabilization and restart during stress conditions^89^.

### The role of BAP1 in DNA replication

The role of BAP1 in DNA replication was discovered during an investigation of the INO80 chromatin-remodeling complex, which functions in DNA replication under both normal and stress conditions^49,104^. A yeast two-hybrid screen identified BAP1 as a binding partner of INO80, the catalytic subunit of the INO80 complex. The study showed that BAP1 deubiquitinated and stabilized INO80, whose cellular level is controlled by the ubiquitin-mediated degradation pathway. Both BAP1 and INO80 bound to replication forks during S phase, and BAP1 depletion inhibited both INO80 recruitment to the fork and fork progression. Therefore, BAP1 promoted replication fork progression via dual mechanisms: stabilization via deubiquitination and recruitment of INO80 to the replication fork. H2Aub was found to be enriched at replication forks, and depletion of RNF2, the major E3 ligase of H2A, inhibited BAP1 recruitment to forks, suggesting that H2Aub mediated BAP1 recruitment^49^. Importantly, a correlation was found between BAP1 and INO80 expression in mesothelioma, and re-expression of BAP1 in H226 cells exhibiting low levels of INO80 fully rescued the INO80 levels, showing that the low INO80 expression level in H226 cells was due to a lack of BAP1-mediated INO80 stabilization^49^. A recent study reported that BAP1 regulates DNA replication in cooperation with the C-terminus of Hsp70-interacting protein (CHIP), which is an E3 ubiquitin ligase of INO80^51^ (Fig. 3).

A subsequent study showed that BAP1 functions in the recovery of replication stress via INO80. BAP1 and INO80 bound to replication forks in response to replication stress induced by nucleotide depletion after hydroxyurea (HU) treatment. BAP1 depletion abolished the increase in INO80–chromatin binding and PCNA-overlapping replication focus formation after HU treatment^50^. RAD51 accumulated at stalled/collapsed forks and promoted both the restart of stalled forks and HR repair of DSBs generated by fork collapse^105^. BAP1 depletion prevented RAD51 from binding to chromatin and forming replication foci after HU treatment. BAP1 depletion also resulted in an increased quantity of stalled forks and in HU sensitivity, which were fully recovered by the ectopic expression of INO80. These results suggest that BAP1 promoted both the restart of stalled forks and cell survival during replication stress via its interaction with INO80^50^ (Fig. 3).

### Unsolved problems and potential directions for future research

Several important issues regarding the role of BAP1 in DNA replication remain unresolved. Although BAP1 promoted fork progression and restart of stalled forks indirectly via its interaction with INO80, direct mechanisms are also possible. For example, since H2Aub is involved in heterochromatin replication^106,107^ and both BAP1 and RNF2 bind to replication forks^49^, BAP1 and RNF2 (in the form of PRC1) may fine-tune H2Aub levels via counteracting activities to regulate chromatin conformation for efficient fork progression and the stalled fork restart (Fig. 3). Given the role of BAP1 in HR repair and RAD51 recruitment^44,46,50^, BAP1 may also contribute to the restart of stalled forks by directly promoting DNA repair at collapsed forks. These possibilities can be tested by using a BAP1 mutant lacking the INO80 interaction motif or in an INO80-null background. Additionally, BAP1 shows dual functions in regulating cell proliferation, depending on the cell type. For example, while re-expression of BAP1 in BAP1-null H226 cells retarded cell cycle progression leading to an accumulation of cells in the S phase^2^, depletion of BAP1 in some BAP1-proficient cells led to reduced cell proliferation with delayed G1-to-S progression^9,29,32,34,49^. Differential regulation of BAP1 in replication fork progression between different cell types might explain these seemingly contradictory outcomes. Hence, it will be of great interest to investigate how the replication-stimulating activity of BAP1 is regulated in cells in which BAP1 suppresses proliferation. The results from these experiments will provide important insights into the recently reported oncogenic roles of BAP1 in certain cancer types^37,108,109^. Furthermore, BAP1 is phosphorylated at Ser592 in response to HU-induced replication stress^45^. The role of BAP1 phosphorylation by ATM and possibly by ATR under replication stress conditions is still unknown and needs to be addressed in future studies. Finally, more studies are needed to determine whether PARP1 functions during unperturbed and stressed DNA replication through its intrinsic and PARylation-mediated activity control of BAP1 (Fig. 3).

## The role of BAP1 in maintaining genome stability

Genome integrity is critical to maintain cellular homeostasis and prevent diseases, such as cancer, and can be threatened by factors that induce genome instability, which is defined as an increased tendency for the genome to acquire mutations, ranging from changes to the nucleotide sequence to chromosome structural abnormalities and aneuploidy (gain or loss of whole chromosomes). The major sources of these mutations are genotoxic insults that cause DNA damage, replication fork collapse and defective mitosis. Malfunction and/or dysregulation in damage repair and mitotic chromosome segregation can lead to genome instability. Unless managed in an efficient and timely manner, genome instability can cause oncogene activation and tumor suppressor loss, which can potentially lead to the development of cancer^110,111^.

BAP1 has been shown to be important for chromosome integrity in various cells from different species. Ablation of *BAP1* in IR-treated DT40 cells resulted in an elevated level of chromosome aberrations, including chromatid/isochromatid breaks and gaps^44^. CRISPR/Cas9-mediated knockout of *BAP1* in PANC1 murine pancreatic cancer cells and HEK 293 T cells induced chromosome abnormalities, including chromosome breaks, shattering and aneuploidy, and these effects were exacerbated by cell exposure to IR^76^. Transient knockdown of BAP1 in HCT116 human colon cancer cells increased chromosome structural abnormalities and aneuploidy in the absence of exogenously induced DNA damage^49^. These results highlight the evolutionarily conserved role of BAP1 in the maintenance of chromosome integrity under both normal and DNA-damaging conditions. These roles of BAP1 are likely attributable to its activities in DNA repair and replication stress recovery as well as chromosome segregation, as discussed below.

Several reports have shown that BAP1 contributes to chromosome integrity by targeting the machinery for spindle assembly and chromosome segregation. γ-Tubulin, a member of the tubulin family, localizes at the centrosome and plays a key role in microtubule nucleation and spindle assembly during mitosis. A study reported that BAP1 interacts directly with and localizes to γ-tubulin during mitosis and that BAP1 stabilizes γ-tubulin via deubiquitination to support microtubule nucleation and mitotic spindle assembly, thereby ensuring chromosome segregation and preventing chromosome abnormalities. This finding is clinically significant because BAP1 is downregulated in metastatic adenocarcinoma breast cell lines, and low expression of BAP1 has been associated with reduced overall survival of breast cancer patients^112^. Other studies have documented that BAP1 targets centrosome proteins. BAP1 interacted with MCRS1 and DID01 and stabilized these centrosome proteins via deubiquitination, which partially contributed to the suppression of multipolar spindle formation and chromosome aberrations in HK-2 human kidney cells. Importantly, a positive correlation between BAP1 and MCRS1/DID01 expression has been identified in ccRCC tissues, and downregulation of MCRS1/DID01 in BAP1-deficient tumors has been associated with adverse clinicopathological features^113,114^.

The association of BAP1 loss with chromosome instability is clearly found in malignant mesothelioma. BAP1-null H226 mesothelioma cells with normal growth exhibited elevated levels of aberrant chromosomes, such as micronuclei and internuclear bridges^44^. Analysis of tumor biopsy samples revealed chromothripsis in malignant mesothelioma, which was caused by chromosome breakage and inaccurate assembly via random inter- or intrachromosomal DNA end-joining repair^115,116^. Because this study screened tumors for somatic copy number loss throughout the 3p21 region, which harbors the BAP1 gene, whether these genetic alterations were caused by BAP1 deletion or occurred independently is unclear^115^. Nonetheless, it is possible that loss of BAP1 DNA repair activity may have accelerated chromothripsis through a positive feedback mechanism. Genetic alterations caused by chromothripsis in mesothelioma were independently confirmed by analysis of primary tumors and matched tumor-derived cell lines^117,118^. These studies additionally found that in mesothelioma cells, chromosomes underwent chromoplexy, a newly discovered type of chromothripsis in which intrachromosomal regions undergo extensive rearrangement^119^. While major causes of chromothripsis are replication stress and mitotic errors, the mechanistic basis of chromoplexy, although poorly characterized to date, is thought to be related to DSBs that are induced by transcription factors binding on open chromatin structure^119^. Since BAP1 is involved in all these processes, it is possible that the loss of BAP1 may be a key contributor to these genetic alterations.

**Fig. 4:**
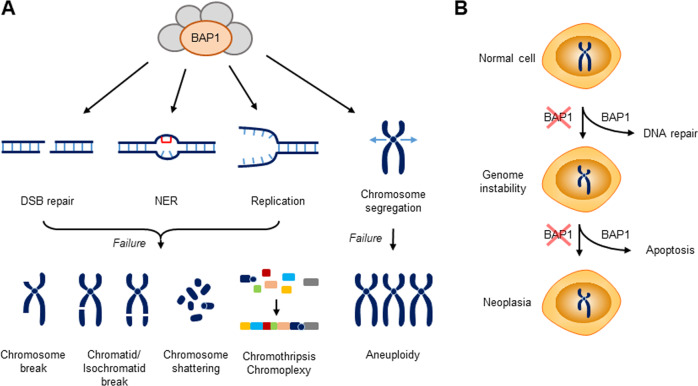
Model showing the roles of BAP1 in genome stability and tumor suppression. **A** BAP1 depletion leads to chromosome structural abnormalities, including chromosome breaks and shattering and chromatid/isochromatid breaks and gaps, in various cell types from different species. Mesothelioma cells carrying inactivating *BAP1* mutations exhibit chromothripsis and chromoplexy, which are caused by chromosome shattering followed by random inter- and intrachromosomal DNA end-joining events. These chromosomal alterations are likely due to loss of BAP1 activity during DNA repair and failed stalled/collapsed replication fork recovery. BAP1 also functions in spindle assembly and chromosome segregation, which prevents aneuploidy. **B** Model showing the role of BAP1 in tumor suppression. BAP1 suppresses genome instability via its DNA repair activity and eliminates cancerous cells that exhibit genome instability by promoting apoptosis. BAP1 loss/inactivation results in a lack of both these genome-stabilizing and cell-elimination activities, which accelerates tumorigenesis, leading to neoplasia.

In summary, BAP1 inactivation leads to various genetic alterations, including chromosome structural abnormalities, aneuploidy and possibly to chromoanagenesis, including chromothripsis and chromoplexy. The contribution of BAP1 loss to the diverse array of genetic alterations likely relies on its roles in many different processes crucial for genome integrity, including DNA damage repair, replication fork progression, collapsed fork restart and chromosome segregation (Fig. 4A). In addition, BAP1 loss/inactivation causes chromosome aberrations in various cell types from different species ranging from chickens to humans, highlighting its essential role in genome stability throughout evolution. Furthermore, clinical data show that the extent of chromoanagenesis is positively correlated with poor prognosis in mesothelioma patients^117^. Therefore, genome instability caused by inactivating *BAP1* mutations may play a key driving role in the tumorigenesis of human cancers associated with BAP1 cancer syndrome.

## Therapy for BAP1-related cancers

Loss-of-function mutations in tumor suppressor genes can be indirectly targeted in a synthetic lethality approach, which relies on the inhibition of pathways on which cancer cells with mutations have become dependent for survival^120^. For example, PARP1 promotes the repair of ssDNA breaks, which, unless repaired, can become DSBs, and PARP inhibitors therefore selectively target cancer cells with defective DSB repair ability and have thus been used to treat breast cancer patients carrying BRCA1 or 2 mutations^75^. The role of BAP1 in DSB repair may provide a therapeutic window for BAP1-defective cancer. This treatment is deemed plausible on the basis of an increase in U2OS and DT40 cell sensitivity to PARP inhibitors after BAP1 depletion^44,46^ and on the 769-P ccRCC cell line carrying an inactivating *BAP1* mutation that makes it more sensitive to IR and the PARP inhibitor olaparib than control cells carrying wild-type *BAP1*^9^. Recent studies have suggested that PARP inhibitors may be effective in treating malignant pleural mesothelioma (MPM), which shows a poor response to current chemotherapy and radiotherapy treatments. MPM cell lines expressing a novel alternative splice isoform of BAP1 and exhibiting reduced DUB activity are more sensitive to olaparib than controls expressing wild-type BAP1^121^. Analysis of MPM cell lines and clinical samples with known HR defects showed that BAP1 loss increased sensitivity to olaparib^122^. However, another study with multiple MPM cell lines showed that PARP inhibitors decreased cell viability and this decrease was accompanied by extensive replication fork collapse and genome instability, regardless of the *BAP1* mutation status^123^. In addition, recent clinical studies have indicated that PARP inhibition did not selectively target BAP1-deficient mesothelioma cells^124–126^. PARP inhibition might have exerted an effect on uncharacterized BAP1-irrelevant DNA repair pathways on which cancer cells rely for survival. Alternatively, the intimate link between BAP1 and PARP1 in the same DNA repair pathway may be a potential cause of the poor clinical outcomes^46,47^. The mechanisms underlying BAP1-independent lethality due to PARP inhibition in mesothelioma cells and possibly other cancer cells remain to be elucidated. Further studies are clearly required to substantiate the applicability of PARP inhibition to a synthetic lethality-based treatment of BAP1-related cancers.

## Concluding remarks

A series of recent studies have documented the roles of BAP1 in DSB repair, NER, replication fork progression, stalled fork restart and chromosome segregation, thereby highlighting a role for this DUB as a guardian of genome stability (Fig. 4A). These roles of BAP1 likely account at least in part for its tumor suppressor function in human cancers associated with BAP1 cancer syndrome. We propose that BAP1 exerts two-level protection against cancer development through its roles in genome stability and apoptosis, the two critical steps that can lead to neoplasia when defective. Specifically, BAP1 suppresses genome instability via its DNA repair activity and eliminates cancer-prone cells that exhibit genome instability by promoting apoptosis. BAP1 loss/inactivation therefore results in a lack of both of these activities, accelerating tumorigenesis, which indicates that this DUB is a powerful tumor suppressor (Fig. 4B).

Many important issues remain to be addressed. It is necessary to examine whether genome instability causes tumorigenesis in cancers that arise by BAP1 loss/inactivation and to what extent the role of BAP1 in genome stability contributes to tumor suppression relative to that of other mechanisms, such as apoptosis. In addition, determination of the mechanisms that underlie BAP1 targeting of many different nuclear processes and how this targeting is regulated in the coordination with cell cycle progression is an urgent research need. From a mechanistic perspective, how BAP1 promotes the repair of DSBs and UV-induced lesions is largely unknown. In particular, since H2Aub is the only known BAP1 substrate at DNA lesions identified thus far, the identification of new BAP1 targets, such as components of repair machinery, warrants a future research focus. Notably, whether BAP1 functions in DNA repair pathways in addition to HR and NER, such as those related to base excision repair and single-strand break repair, is an outstanding question and is relevant because PARP1 plays an important role in these pathways. Resolving all these compelling issues will provide a better picture of how BAP1 prevents cancer as a guardian of the genome.
